# Health technology assessment (HTA) readiness in Uganda: stakeholder’s perceptions on the potential application of HTA to support national universal health coverage efforts

**DOI:** 10.1017/S0266462323002635

**Published:** 2023-10-31

**Authors:** Chrispus Mayora, Joseph Kazibwe, Richard Ssempala, Brenda Nakimuli, Aloysius Ssennyonjo, Elizabeth Ekirapa, Sarah Byakika, Tom Aliti, Timothy Musila, Mohamed Gad, Anna Vassall, Francis Ruiz, Freddie Ssengooba

**Affiliations:** 1 Department of Health Policy Planning and Management, Makerere University School of Public Health, Kampala, Uganda; 2 Department of Global Health and Development, London School of Hygiene and Tropical Medicine, London, UK; 3 Department of Economic Theory and Analysis, Makerere University School of Economics, Kampala, Uganda; 4 Department of Planning, Financing and Policy, Ministry of Health, Kampala, Uganda

**Keywords:** health technology assessment (HTA), perception and experiences, Uganda, decision making, priority setting, low and middle income countries

## Abstract

**Introduction:**

Health technology assessment (HTA) is an area that remains less implemented in low- and lower middle-income countries. The aim of the study is to understand the perceptions of stakeholders in Uganda toward HTA and its role in decision making, in order to inform its potential implementation in the country.

**Methods:**

The study takes a cross-sectional mixed methods approach, utilizing an adapted version of the International Decision Support Initiative questionnaire with both semi-structured and open-ended questions. We interviewed thirty key informants from different stakeholder institutions in Uganda that support policy and decision making in the health sector.

**Results:**

All participants perceived HTA as an important tool for decision making. Allocative efficiency was regarded as the most important use of HTA receiving the highest average score (8.8 out of 10), followed by quality of healthcare (7.8/10), transparency (7.6/10), budget control (7.5/10), and equity (6.5/10). There was concern that some of the uses of HTA may not be achieved in reality if there was political interference during the HTA process. The study participants identified development partners as the most likely potential users of HTA (66.7 percent of participants), followed by Ministry of Health (43.3 percent).

**Conclusion:**

Interviewed stakeholders in Uganda viewed the role of HTA positively, suggesting that there exists a promising environment for the establishment and operationalization of HTA as a tool for decision making within the health sector. However, sustainable development and application of HTA in Uganda will require adequate capacity both to undertake HTAs and to support their use and uptake.

## Introduction

Priority setting is a key aspect of attaining universal health coverage (UHC) (1). UHC is commonly interpreted as people and communities receiving the health services they need without experiencing financial hardship, no matter who they are or where they are (2). Many low- and middle-income countries (LMICs) are aiming to achieve UHC by 2030, introducing reforms, for example, to remove financial barriers to care and eradicate the financial burden to the patients and their households. Some of the countries enacting UHC-relevant reforms include among others, Vietnam (3), China (4), Ghana (5), Uganda (6), and Zambia (7).

Many UHC reforms in LMICs are focused at increasing the available resources for the health system while reducing out of pocket expenditure on health (8). This leaves out the element of how the resources are spent, the purchasing function of the health financing building block of the health system. This includes the process by which funding priorities are made (priority setting for health). As countries grapple with increasing resource constraints with many emphasizing the need to maximize value for money, current institutional structures are often inadequate to support the efficient allocation and use of available resources (9). Most decisions (including priority setting decisions) are made in *ad hoc* and implicit manner (10;11). However, some countries have made progress in this space through incorporating evidence-informed decision making (EIDM) approaches in health policies as a means to effectively and efficiently respond to the health needs of served populations. The World Health Organization (WHO) defines EIDM as a systematic and transparent approach that applies structured and replicable methods to identify, appraise, and make use of evidence across decision-making processes, including for implementation (12).

The WHO asserts that priority setting decisions should be informed by the best available evidence from research, as well as other factors such as context, public opinion, equity, feasibility of implementation, affordability, sustainability, and acceptability to stakeholders (12). Countries in the global south have increasingly introduced EIDM mechanisms such as health technology assessment (HTA); or at least expressed a desire to do so (13–15).

HTA has been defined as a multidisciplinary process that uses explicit methods to determine the value of a health technology at different points in its lifecycle (16). The purpose is to inform decision making in order to promote an equitable, efficient, and high-quality health system (17). HTA has been highlighted by the WHO as one of the tools that can propel countries toward UHC if implemented. Examples of countries recently introducing HTA in decision making include Ghana (15;18), India (19), Kenya (20), Indonesia (21), and the Philippines (22). Uganda is committed to the achievement of UHC by 2030 through strengthening the health system and its support mechanisms with a specific focus on primary health care (23). The Ministry of Health (MoH) sees HTA as one of the tools that can enable the country make further progress toward UHC.

Uganda, a country located in East Africa, is a low-income country with a gross domestic product per capita of USD 883.9 (24) and a population of 47 million as of 2021 (25). The Ugandan health system is decentralized from MoH to the districts but with the financing still largely controlled by the central government, there is little allocative authority available to the districts. The MoH is responsible for policy formulation, planning, quality assurance, epidemic response, international relations, resource mobilization and monitoring, and evaluation. Most decision making takes place at the ministry level. Resource allocation at national level is done every financial year by the Ministry of Finance Planning and Economic Development (MoFPED) to different sectors, that is, ministries, departments, and agencies (MDAs) like MoH based on the government priorities and fiscal space. Each MDA is then required to prepare a budget framework paper attaching the allocated resources to specific program outputs and activities.

The budgetary allocations to the health sector have been increasing over time from 560 billion Uganda Shillings (UGX) (USD 219.2 million) in the financial year (FY) 2010/11 to 911 billion UGX in 2018/19 (USD 244.4 million) (26). However, the MoH budget as a share of the total government budget has been showing a downward trend even before COVID-19 pandemic. The budget declined from 11.2 percent in 2004/5 to 5.1 percent in 2020/21 (27). There was an increase to 7.5 percent for FY 2021/22 after the start of COVID-19 (28). Out-of-pocket expenditure on health as a proportion of the national current health expenditure remains high at 40 percent as of FY 2018/19 (29;30).

Uganda has a total of 6,937 health facilities and clinics at different levels in 128 districts (31). The biggest share of all health facilities is government owned at 45.16 percent (3,133); another 14.44 percent (1,002) are private and not-for-profit (PNFP), 40.29 percent (2,795) are private for profit (PFP), and 0.10 percent (7) community-owned facilities. Uganda has made improvements in healthcare coverage in recent years. The Government of Uganda planned to have at least 85 percent of the population living within 5 kilometers of a health facility by 2020 from 75 percent as of 2015 (32). This target, however, has not yet been achieved (33).

The International Decision Support Initiative (iDSI), a global network of priority setting support institutions and experts, received a request from the Ugandan MoH to facilitate the institutionalization of HTA in the country (34). Institutionalization of HTA involves the establishment and operationalization of HTA structures and processes that enable the sustainable production and utilization of HTA evidence. Following a literature review, we did prior to this study; we did not find any evidence of past HTA efforts at national level.

As an initial step, and to inform any strategy to implement HTA in Uganda, a situational (or landscape) analysis that examines existing priority setting approaches and the potential capacity to use evidence in decision making was considered necessary. The present study, a component of a wider situational analysis, aimed to understand the perceptions of stakeholders in Uganda toward HTA to inform HTA implementation in the country.

## Methods

### Study design

This is a cross-sectional mixed methods study utilizing a questionnaire with both semi-structured and open-ended questions.

### Data collection tool

The iDSI HTA situational analysis survey questionnaire (35) was used to collect data from key informants. The questionnaire was selected because it has previously been used in a survey of sub-Saharan African countries and also in a more in depth study focused on Nigeria (36). The questionnaire was adapted to the Uganda setting and used to collect the primary data. Two forms of adaptation were carried out, adaptation to improve comprehension and adaptation to improve coverage. The adaptation to improve comprehension involved replacing general terms with specific terms for example changing “your country” to “Uganda.” Adaptation to improve coverage involved making additions to original/source questions to get more specific answers from the respondents such as asking the respondents to give examples. No language translations were done. The adaptation of the tool was done during a 1-day virtual workshop organized by the research team consisting of members from Makerere University School of Public Health (MakSPH), MoH, and iDSI (co-authors on this study). The questionnaire is divided into two parts: a closed ended (quantitative) series of questions focused on the uses and importance of HTA; and an open-ended questionnaire section that seeks information on the need, demand and supply of HTA. The quantitative sections score the importance of HTA in a number of dimensions: achieving allocative efficiency; quality of care; transparency in decision making; budget control; and equity. The scores ranged from 0 to 10, where 0 is “not important” and 10 is “very important.” The key informants were expected to answer both the quantitative and qualitative sections of the questionnaire. The questionnaire was pretested among five non-key informants at the MoH to ensure that the questions were clear and uniformly understood by the people answering them.

### Study participants

Participants were people in decision-making positions within their organizations or institutions. A total of thirty-three key informants were identified and invited to participate in the study. They were purposively selected to represent a variety of stakeholder institutions involved in policy making in the health sector which are likely to utilize and supply HTA evidence. The selection of the key informants was based on expertise, experience, and role in the decision making and resource-allocation/prioritization processes in the health sector at both regional and national level. The key informants were identified through a rapid literature review, and consultations with the MoH. The key informants included: representatives of the professional councils; district health officers; development partners; government institutions such as the National Drug Authority; investigators at academic and research institutions; development partners and non-governmental organizations (all of which were civil society organizations, CSOs). The interviews took place between January and March 2022.

### Administration of the questionnaire

Appointments were made with the participants prior to the interview day. The interviews were conducted either in person or virtually via Zoom taking into account the key informant’s preference and the existing COVID 19 guidelines in the country at that time. The questionnaire was interviewer-administered. All interviews were recorded. In addition, the interviewer took notes during the interview which were handed over to the principal investigator after the interview for referencing during analysis.

### Data processing and analysis

The recorded interviews were transcribed. The quantitative data were cleaned and descriptively analyzed using Microsoft Excel. The data were summarized narratively in addition to using tables and graphs.

The qualitative data were analyzed using the inductive thematic analysis method (37) taking the constructionist thematic approach. This involved reading the transcripts several times to identify meaningful statements that were coded. The codes were then categorized. The meaningful statements were reported to provide context and explanation to the quantitative data. Validation of results was done through a meeting with the stakeholders.

### Ethical considerations

This study was reviewed and approved by the MakSPH Higher Degrees Research and Ethics Committee (HDREC), approval number SPH-2021-151. The study was registered with the Uganda National Council of Science and Technology. Ethical approval was also sought from and granted by the London School of Hygiene and Tropical Medicine Ethics Committee (LSHTM Ethics Ref: 26615). During data collection, ethical principles were upheld in the study including: (i) maintaining the level of confidentiality; (ii) informant’s participation was voluntary; (iii) obtaining written informed consent; and (iv) no study materials contained names or other explicit identifiers of participants.

## Results

A total of thirty stakeholders from governmental, non-governmental, and private sector institutions were interviewed to understand their perceptions toward the use of HTA in decision making. Supplementary Table S1 shows the list of institutions from which the key informants were selected.

Proportionately, the key informants included in this study reflected a balanced representation of the supply and demand side of HTA in the country. The supply side of HTA was mostly represented by academic institutions (*n* = 5) while the majority of key informants on the demand side were government ministries (*n* = 8).

The results are presented under the following four themes based on the analyzed data: (a) perceived use of HTA, (b) areas that require HTA urgently, (c) potential users of HTA outputs, and (d) perceived level and type of HTA evidence needed by major stakeholders.

### Perceived use of HTA

**Figure 1.**
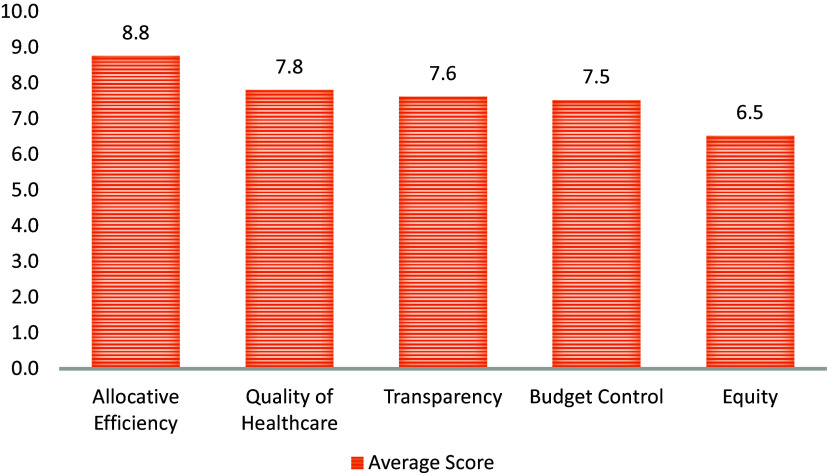
Perceived importance of HTA among stakeholders.

The stakeholders perceived achieving allocative efficiency, quality of care, transparency in decision making, budget control, and equity as key uses of HTA. Each of these aspects had average scores greater than 6 out of 10 (where 10 indicates the use is “very important”). Allocative efficiency was scored as the most important use of HTA gaining an average score of 8.8. Figure 1, a graph showing the average scores awarded to each of the HTA benefits.

Value for money and optimal use of resources were common reasons provided for scoring allocative efficiency highly. HTAs were viewed by many as a way of getting the best value from the available resources enhancing the efficiency of the health system. For example, one of the key informants mentioned that there was a mismatch between the amount of resources allocated to the different levels of health facilities and the services the health facilities are expected to provide. The informant indicated that HTA would help prevent such scenarios.

*“Health center IV, we give resources in the same way that we give… other lower-level facilities, and yet you know very well that almost 30 percent of these health centers are not carrying out operations but they are getting money like the health center IVs which are having like sixty or fifty operations per month. So, there you are not allocating your resources efficiently. There is no allocative efficiency in that aspect. So, it means that you need to have that information and you need to develop that criterion, the whole of that formula then you can be able to say that you can efficiently allocate, we must be conscious of what we are doing….”* Key informant (K3)

Others emphasized the need for transparency in decision making to ensure that the resource allocation is aligned with the main problems from the communities’ perspective. A key reason for adopting HTA was that it would help improve the quality of health care.

*“Improving the quality of healthcare, …… requires having in place tools that can help you to continuously assess and identify the gap on a regular basis. So, if you go manual, you cannot get all the information and analyze it in time and triangulate and give you a good assessment of the different perspectives of quality…. HTA can assist in ensuring provision of quality services.”* Key informant (K9)

*“Why would I give it [improving quality of healthcare] a higher score… because interventions implemented based on evidence are less prone to embezzlement. …, resources are put to their best use.”* Key informant (K23)

Despite a high score on the *need* for HTA, many respondents acknowledged that there could be a less-than-optimal use of HTA in actual decision making due to political pressures and other considerations. For example, limited resources and capacity may not support the actual use of evidence in practice.

*“I will give it about 8 (allocative efficiency), because as much as you can have the assessment [HTA] done, …when it comes to decision making, there are other factors that also influence things like political decisions, interests and all that. It is good, but you cannot rely on it 100 percent.* *”* Key informant (K9)

### Areas that require HTA urgently

**Figure 2.**
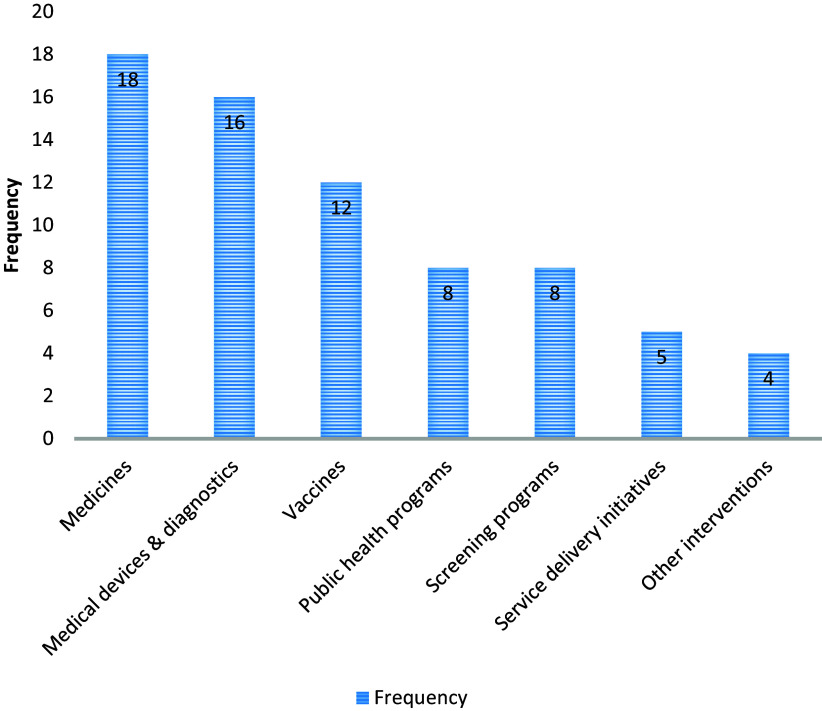
Areas that require the use of HTA urgently.

Each respondent was asked to identify three technology (or intervention) areas that they thought urgently required HTA. A number of areas were identified including medicines, medical devices and diagnostics, public health programs, vaccines, screening programs, and service delivery incentives (Figure 2).

The assessment of medicines was identified as the area HTA was most urgently needed (eighteen respondents (60.0 percent)), followed by medical devices and diagnostics (sixteen respondents (53.3 percent)), and vaccines (twelve respondents (40.0 percent)) in third place (Figure 2).

Most respondents highlighted that these technology areas are expensive relative to the limited available resources. Other issues noted by respondents included the use of expensive first line drugs by Ugandan health providers, especially in the private sector, despite the availability of more affordable alternatives.

*“… these are things (medicines, vaccines, and medical devices) which are common in a health system service delivery and because they are there, we interface with them whether you are a patient or not. […] The diagnostic devices [….], people are taken advantage of with these devices so I think to help the user, these need to go through HTA process and government or the country makes a statement on the various diagnostics; then the private sector is unlikely to take advantage of the population …….”* Key informant (K10)

### Potential users of HTA outputs

**Figure 3.**
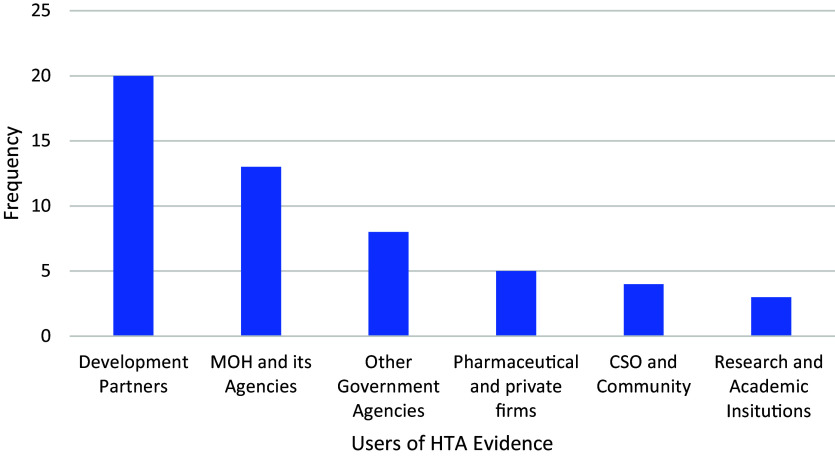
Likely users of HTA evidence.

Respondents were asked for the likely users of HTA outputs. Figure 3 shows the frequency of perceived likely users of HTA outputs in Uganda.

Development partners were perceived to be most likely users of HTA output (twenty of thirty respondents) followed by the MoH and its subsidiaries (thirteen of thirty). Only five respondents perceived pharmaceutical and private firms as potential users of HTA outputs, while CSOs, community and research institutions were seen as potential users of HTA output by less than five respondents.

“*Donors are good consumers of this data (HTA evidence); it can help with allocating the limited resources that are usually available. They focus more on the top priority.”* K8

### Perceived level and type of HTA evidence needed by major stakeholders

**Figure 4.**
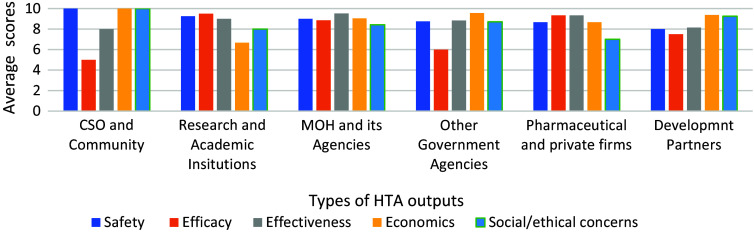
Perceived need for the different types of HTA outputs by stakeholders.

Respondents scored the extent to which the different type of HTA outputs are needed by the major categories of stakeholders (Figure 4).

All the different potential HTA outputs (relating to safety, efficacy, effectiveness, economic and social/ethical concerns) are seen to be needed and helpful to all categories of stakeholders (Table 1). All the outputs were scored at least five out of ten for each category of stakeholders. ‘Efficacy’ was scored at 5/10 for the CSO and community stakeholders.

**Table 1.** Level of interest in different types of HTA outputs

Organization	Safety	Efficacy	Effectiveness	Economics	Social/ethical concerns
Development partners	8.00	7.50	8.14	9.37	9.25
MOH and its agencies (NMS, UNITAG, NDA)	9.00	8.85	9.53	9.06	8.41
Other government agencies (MoFPED, NPA, KCCA, and UBOS)	8.75	6.00	8.83	9.57	8.71
Pharmaceutical and private firms (JMS)	8.67	9.33	9.33	8.67	7.00
CSO and community	10.00	5.00	8.00	10.00	10.00
Research and academic institutions	9.25	9.50	9.00	6.67	8.00

*Source*: Averaged from respondent scores.

The CSO category of stakeholders was perceived to be the most interested in safety-related outputs of HTA (10.00/10) followed by research and academic institutions (9.25/10), and the MoH and its subsidiaries (9.00/10). Research and academic institutions were identified as the most interested in efficacy aspects (9.50/10) followed by pharmaceutical and private firms (9.33/10). The MoH and its subsidiaries was identified as the stakeholder most interested in effectiveness outputs of HTA (9.53/10) followed by pharmaceutical and private firms (9.33/10). The CSO were perceived to be the most interested in economic-related HTA outputs (10 out of 10), followed by development partners (9.37/10). The ranking of the interest of the stakeholders in social/ethical concerns was similar to the interest in economic evidence.

## Discussion

This study shows that HTA stakeholders (decision makers) on both the supply and demand sides of HTA perceive HTA as an important tool for decision making within the health sector in Uganda. According to this study, seeking allocative efficiency is the most important goal when implementing HTA, and medicines were identified as the main technology area where the application of HTA type analyses were long overdue. Notably, development partners were perceived to be the most likely users of HTA outputs. To the best of our knowledge, this is the first HTA situational analysis carried out in Uganda.

Resource allocation was perceived as the most prominent use of HTA according to the stakeholders in Uganda. This is consistent with findings in India (35) and Nigeria (36) where similar surveys have been done. HTA has been taken to be synonymous with cost effectiveness (38) and limited to the end result of resource allocation or some assessment of value-for-money. However, HTA arguably offers further benefits in terms of transparency, support for equity considerations and stakeholder inclusiveness, as part of an overarching priority setting decision process (39–41). HTA is not a narrow technical exercise; it provides a framework to accommodate multiple considerations/criteria including potentially “political factors” within a multi-stakeholder engagement process. A good HTA process follows pre-agreed rules and offers transparency with respect to how any evidence is considered in decision making. These factors enhance the credibility and social legitimacy of often difficult priority setting choices (42).

Development partners were identified as the most likely users of HTA in Uganda in this study which differs from the findings of similar studies in other countries. In Nigeria and India, it was found that that federal and state governments were seen as the dominant users of HTA outputs (35;36). This difference could reflect variation in development partner influence in resource allocation decisions in these settings. In both Nigeria and India, the state governments have extensive authority to make such decisions, while in Uganda, most of the healthcare resources have been ear-marked already, and to a significant extent these ear-marked resources are supported by funding from development partners. Indeed, development partners fund approximately 40 percent of the Ugandan health budget (30). The findings from Uganda may also reflect the stakeholders’ association of HTA with primarily the allocation of resources for specific technologies, with less consideration of other uses such as informing the development of clinical guidelines. In addition, the perception that the development partners are the most likely users of HTA raises some concerns about the sustainability of implementation of HTA in the country. As the country progresses toward middle-income status, development assistance may concomitantly fall. In the event that the perceptions do not change, there is a risk that, counter-intuitively, there may be a reduced HTA supply hindering institutionalization in the country. On the other hand, reduced external support may lead to a greater focus on ensuring value for money for domestically allocated budgets given increasing country responsibility for their management. As such HTA demand from policy makers may actually increase and help drive domestic supply. Further sensitization of Ugandan stakeholders on the nature and use of HTA may change perceptions around key beneficiaries of implementation.

It is perhaps concerning that the MoH is not seen as the most likely user of HTA evidence despite the fact that it is the institution responsible for policy formulation and implementation within the health sector. This may in part be a result of the relatively low engagement of stakeholders by the MoH on the topic of HTA. This suggests that there could be value in the MoH (with the support of international partners as needed) in actively engaging with relevant stakeholders, and advocate for a potential legal framework to guide the operationalization and institutionalization of HTA in the country. Building HTA within a legal framework that requires it use for coverage decisions could be valuable in aiding implementation, especially in environments where there is a national social insurer (43). For example, the Philippines put in place an HTA organizational structure informed by a statutory law that established the Health Technology Assessment Council (HTAC), an independent advisory institution which gives guidance to the Philippines Government Department of Health and the Philippine Health Insurance Corporation (PhilHealth) on which health interventions/technologies are to be funded by the government (22).

Uganda, through the MoH, is in fact pursuing a legal framework detailing the processes through which priority setting decisions are made and operationalized. However, significant progress can still be made in the absence of a detailed legal underpinning for HTA. For example, Thailand has developed robust HTA systems without a specific legal framework and has created a semi-autonomous unit to serve as an agency to inform decisions using HTA (44). Rwanda, while not having a dedicated HTA unit, is currently applying HTA approaches to update the health benefits package of the community-based health insurance scheme (45). Uganda could therefore explore setting up preliminary, exploratory structures to support HTA development, such as an HTA unit perhaps within the MoH, in advance of a formal legal framework. This would allow for testing potential options and processes.

The relative absence of HTA-like approaches in decision making in Uganda was seen by the stakeholders interviewed as disadvantaging the patients and people in Uganda in general. The cost of medicines is not regulated, and patients are seen as being taken advantage of. This potentially exposes patients and their households to catastrophic health expenditures. It is for these reasons that respondents see HTA as a tool to support development of the national drug formulary, standardize reimbursement of expenses especially for provider payment systems and improve health outcomes of the final users.

The establishment of HTA structures is likely to stimulate and increase HTA capacity in the country. Currently, there is a limited HTA relevant literature that focuses on the Ugandan context, and the majority of those studies are authored by researchers based in other countries, a situation similar to LMICs more generally (46). The existence of HTA structures will further enhance the value of the awaited Master of Health Economics program by Makerere University which will train health economists in-country that could then be absorbed into those HTA structures. Makerere University has developed a curriculum for the master’s program which has been approved by the Makerere University Council. It should be noted that universities play an active role in the establishment of HTA structures in the country and are crucial elements in capacity building and in supporting the supply of locally generated assessments (47). For example, universities have been crucial in carrying out training on HTA in Kenya (48) and India (49).

Building in-country HTA structures will, however, still need the support of international organizations and donors for the foreseeable future. There are a number of development partners that are engaging in HTA relevant activities within the country including the Medical Research Council/Uganda Virus Research Institute and LSHTM Uganda Research Unit, the University of York under Thanzi la Onse, the Professional Society for Health Economics and Outcomes Research (ISPOR), Results for Development (R4D), ThinkWell, Strategic Purchasing Africa Resource Centre (SPARC), Norwegian Institute of Public Health (NiPH), and KEMRI-Wellcome Trust. Although important for supporting domestic HTA institutionalization, in most cases, development partners tend to carry out activities in isolation leading to duplication and unnecessary competition if unregulated within a given country. There is therefore a need for the MoH to encourage collaboration and coordination among these stakeholders to avoid duplication of HTA-related activities and optimize impact.

## Next steps

This study provides an overview of the HTA landscape in Uganda, setting out the existing perceptions of different stakeholders on its role in priority setting. It represents initial foundational work to inform a detailed HTA strategy that will describe the capacity strengthening needs, the role of stakeholders, and the path to establishing and operationalizing preliminary HTA structures in Uganda. Supported by development partners and under the leadership of Ugandan authorities, it may be necessary to undertake a detailed capacity assessment exercise in order to better understand existing strengths and where current expertise is located. This would inform a national strategy for capacity building in HTA going forward.

## Limitations

The study included respondents that were in decision-making positions within their organizations. The findings may not reflect mid-level and low-level managers within those institutions. The stakeholders selected were mostly from the central region of Uganda, which is generally urban and houses the headquarters of MoH and most key institutions. Therefore, the findings of this study may not reflect the perceptions of decision makers that are based in rural parts of the country. The level of understanding of HTA varied across stakeholders, where those with a background in health economics having more knowledge on the subject matter than others. This may have introduced some level of bias in the findings. Furthermore, although we aimed to include a diverse assortment of stakeholders, patient representatives were not included in this study despite efforts to reach out to them. They were unavailable at a time. In addition, representation from private industry was limited with only one person included in the study.

## Conclusion

Key stakeholders in Uganda took a positive view of the role of HTA suggesting a promising environment for the establishment and operationalization of HTA as a tool for decision making within the health sector. The perception of stakeholders that HTA output/evidence is most likely to be used by development partners calls for empowerment of the different stakeholders to stimulate local demand, ownership, and use of HTA evidence with MoH taking on the leadership role with support from other stakeholders. Sustainable development and application of HTA in Uganda will require adequate capacity both to undertake HTAs and to support their use and uptake. There is perhaps a need for a more comprehensive understanding of current HTA capacity in Uganda, which takes into account the different needs and requirements of the stakeholders involved, the existing priority setting processes and notes the importance of strengthening both technical and non-technical (e.g., administrative) aspects necessary for the conduct of HTA.

## Supporting information

Mayora et al. supplementary material Mayora et al. supplementary material

## Data Availability

The data analyzed during the current study are available from the corresponding author upon reasonable request.

## Supplementary material

The supplementary material for this article can be found at https://doi.org/10.1017/S0266462323002635.

## References

[1] WHO Consultative Group on Equity and Universal Health Coverage. Making fair choices on the path to universal health coverage. 2014. Available from: http://www.ncbi.nlm.nih.gov/pubmed/25666865 (accessed 2 September 2022).

[2] World Health Organisation. Universal health coverage (UHC). 2021. Available from: https://www.who.int/news-room/fact-sheets/detail/universal-health-coverage-(uhc) (accessed 12 June 2022).

[3] Tran Van T, Thi Phuong H, Mathauer I, Thi Kim Phuong N. A health financing review of Vietnam with a focus on social health insurance: Bottlenecks in institutional design and organizational practice of health financing and options to accelerate progress towards universal coverage. 2011. Available from: https://iris.who.int/handle/10665/341160. (accessed 26 January 2023).

[4] Zhang J, Zeng H, Yu T, Zhang R. China’s universal healthcare reform: The first phase [2009–2011] of the ambitious plan. J Hosp Manag Heal Policy. 2018;2:22. 10.21037/JHMHP.2018.04.09

[5] Fusheini A. Healthcare financing reforms: Ghana’s national health insurance. *Heal Reforms Across World.* March 2020:25–54. 10.1142/9789811208928_0002

[6] Cashin C, Dossou J-P. Can national health insurance pave the way to universal health coverage in sub-Saharan Africa? Health Syst Reform. 2021;7(1):e2006122. 10.1080/23288604.2021.2006122 34965364

[7] Masiye F, Chansa C. Health financing in Zambia. Washington, DC: World Bank; 2019.

[8] Jalali FS, Bikineh P, Delavari S. Strategies for reducing out of pocket payments in the health system: A scoping review. Cost Eff Resour Alloc. 2021;19(1):1–22. 10.1186/S12962-021-00301-8/TABLES/5 34348717 PMC8336090

[9] Glassman A, Chalkidou K, Giedion U, et al. Priority-setting institutions in health: Recommendations from a Center for Global Development Working Group. Glob Heart. 2012;7(1):13–34. 10.1016/J.GHEART.2012.01.007 25691165

[10] Baltussen R, Niessen L. Priority setting of health interventions: The need for multi-criteria decision analysis. Cost Eff Resour Alloc. 2006;4(1):1–9. 10.1186/1478-7547-4-14/FIGURES/2 16923181 PMC1560167

[11] Chalkidou K, Levine R, Dillon A. Helping poorer countries make locally informed health decisions. BMJ. 2010;341(7767):284–286. 10.1136/BMJ.C3651 20639295

[12] World Health Organsiation. Evidence, policy, impact: WHO guide for evidence-informed decision making. 2022. Available from: https://www.who.int/publications/i/item/9789240039872 (accessed 14 December 2022).

[13] Downey LE, Mehndiratta A, Grover A, et al. Institutionalising health technology assessment: Establishing the Medical Technology Assessment Board in India. BMJ Glob Heal. 2017;2(2):e000259. 10.1136/BMJGH-2016-000259 PMC5717947 29225927

[14] MacQuilkan K, Baker P, Downey L, et al. Strengthening health technology assessment systems in the global south: A comparative analysis of the HTA journeys of China, India and South Africa. Glob Health Action. 2018;11(1):1527556. 10.1080/16549716.2018.1527556/SUPPL_FILE/ZGHA_A_1527556_SM6628.ZIP 30326795 PMC6197020

[15] Groom G. Final report: iDSI learning review: Ghana. F1000Research 2019 8840. 2019;8:840. 10.7490/F1000RESEARCH.1116868.1

[16] World Health Organisation. International HTA networks. Available from: https://www.who.int/health-technology-assessment/networks/en/ (accessed 15 April 2021).

[17] O’Rourke B, Oortwijn W, Schuller T. The new definition of health technology assessment: A milestone in international collaboration. Int J Technol Assess Health Care. 2020;36(3):187–190. 10.1017/S0266462320000215 32398176

[18] Bidonde J, Meneses-Echavez JF, Asare B, et al. Developing a tool to assess the skills to perform a health technology assessment. BMC Med Res Methodol. 2022;22(1):78. 10.1186/S12874-022-01562-4/FIGURES/3 35313812 PMC8939100

[19] Ministry of Health and Family Welfare Department of Health Research India. Health technology assessment in India (HTAIn). 2018. Available from: https://htain.icmr.org.in/ (accessed 30 April 2023).

[20] Ministry of Health. Government launches health technology assessment to inform policy decision making Nairobi, Kenya. March 2018. Available from: https://www.health.go.ke/government-launches-health-technology-assessment-to-inform-policy-decision-making-nairobi-kenya-18-march-2018/ (accessed 30 April 2023).

[21] Sharma M, Teerawattananon Y, Luz A, Li R, Rattanavipapong W, Dabak S. Institutionalizing evidence-informed priority setting for universal health coverage: Lessons from Indonesia. Inq (United States). 2020;57:46958020924920. 10.1177/0046958020924920 PMC7285939 32513029

[22] Republic of Philippines Department of Health. Health Technology Assessment Council (HTAC). Available from: https://hta.doh.gov.ph/health-technology-assessment-council-htac/ (accessed 30 April 2023).

[23] Ministry of Health. Ministry of Health strategic plan 2020/21–2024/25. Ministry of Health | Government of Uganda. 2020. Available from: https://www.health.go.ug/cause/ministry-of-health-strategic-plan-2020-21-2024-25/ (accessed 2 September 2022).

[24] The World Bank. GDP per capita (current US$) - Uganda. 2022. Available from: https://data.worldbank.org/indicator/NY.GDP.PCAP.CD?locations=UG (accessed 30 April 2023).

[25] The World Bank. Uganda. 2022. Available from: https://data.worldbank.org/country/UG (accessed 14 December 2022).

[26] Ministry of Finance Planning and Economic Development. Good governance; a prerequisite to harness the demographic dividend for sustainable development. Kampala: Minsitry of Finance Planning and Economic Development; State of Uganda Population Report 2018. 2018.

[27] UNICEF. The national budget framework paper 2020/21 budget brief. UNICEF; 2020.

[28] UNICEF. Safeguarding public investments in the health in the advent of COVID-19. 2022.

[29] Kadowa I. A case study of the Uganda national minimum healthcare package the role of essential health benefits in the delivery of integrated services: Learning from practice in east and southern Africa. Kampala: Ministry of Health Uganda; 2017.

[30] Ministry of Health Uganda. National health accounts 2016–2019. 2022. Available from: https://www.health.go.ug/cause/national-health-accounts-2016-2019/ (accessed 30 April 2023).

[31] Ministry of Health of the Republic of Uganda. National health facility master list. Kampala: Ministry of Health Uganda; 2018.

[32] Ministry of Health Uganda. Health sector development plan 2015/16–2019/20. 2015. Available from: https://www.health.go.ug/cause/health-sector-development-plan-2015-16-2019-20/ (accessed 8 January 2022).

[33] World Health Organisation African Region. Uganda on the right path to achieving universal health coverage. 2018; Available from: https://www.afro.who.int/news/uganda-right-path-achieving-universal-health-coverage (accessed 30 April 2023).

[34] International Decision Support Initiative (iDSI). iDSI. 2020; Available from: https://www.idsihealth.org/ (accessed 6 September 2022).

[35] Dabak SV, Pilasant S, Mehndiratta A, et al. Budgeting for a billion: Applying health technology assessment (HTA) for universal health coverage in India. Heal Res Policy Syst. 2018;16(1):1–7. 10.1186/S12961-018-0378-X/TABLES/2 PMC6262968 30486827

[36] Uzochukwu BSC, Okeke C, O’Brien N, Ruiz F, Sombie I, Hollingworth S. Health technology assessment and priority setting for universal health coverage: A qualitative study of stakeholders’ capacity, needs, policy areas of demand and perspectives in Nigeria. Global Health. 2020;16(1):1–11. 10.1186/S12992-020-00583-2/TABLES/6 32641066 PMC7346669

[37] Braun V, Clarke V. Using thematic analysis in psychology. Qual Res Psychol. 2006;3(2):77–101.

[38] Teerawattananon Y, Painter C, Dabak S, et al. Avoiding health technology assessment: A global survey of reasons for not using health technology assessment in decision making. Cost Eff Resour Alloc. 2021;19(1):1–8. 10.1186/S12962-021-00308-1/FIGURES/3 34551780 PMC8456560

[39] Pan American Health Organisation and World Health Organisation. Health technology assessment (HTA). 2013. Available from: https://www3.paho.org/hq/index.php?option=com_content&view=article&id=9229:2013-tecnologias-sanitarias&Itemid=41687&lang=en#gsc.tab=0 (accessed 30 April 2023).

[40] Gagnon MP, Desmartis M, Lepage-Savary D, et al. Introducing patients’ and the public’s perspectives to health technology assessment: A systematic review of international experiences. Int J Technol Assess Health Care. 2011;27(1):31–42. 10.1017/S0266462310001315 21262085

[41] Gagnon MP, Tantchou Dipankui M, Poder TG, Payne-Gagnon J, Mbemba G, Beretta V. Patient and public involvement in health technology assessment: Update of a systematic review of international experiences. Int J Technol Assess Health Care. 2021;37(1):e36. 10.1017/S0266462321000064 33541449

[42] Rocchi A, Chabot I, Glennie J. Evolution of health technology assessment: Best practices of the pan-Canadian oncology drug review. Clin Outcomes Res. 2015;7:287–298. 10.2147/CEOR.S82549 PMC4461134 26082654

[43] Bertram M, Dhaene G, Edejer TT-T. Institutionalizing health technology assessment mechanisms: A how to guide. 2021. Available from: https://apps.who.int/iris/handle/10665/340722 (accessed 13 May 2023).

[44] Sharma M, Teerawattananon Y, Dabak SV, et al. A landscape analysis of health technology assessment capacity in the Association of South-East Asian Nations region. Heal Res Policy Syst. 2021;19(1):1–13. 10.1186/S12961-020-00647-0/FIGURES/3 PMC7879649 33573676

[45] Government of Rwanda. Rwanda ministerial instructions determining the methodology to define the community-based health insurance benefit package. 2021. Available from: https://gazettes.africa/archive/rw/2021/rw-government-gazette-dated-2021-08-31-no-special.pdf (accessed 27 April 2022).

[46] Hollingworth S, Fenny AP, Yu SY, Ruiz F, Chalkidou K. Health technology assessment in sub-Saharan Africa: A descriptive analysis and narrative synthesis. Cost Eff Resour Alloc. 2021;19(1):1–13. 10.1186/S12962-021-00293-5/TABLES/2 34233710 PMC8261797

[47] Fontrier AM, Visintin E, Kanavos P. Similarities and differences in health technology assessment systems and implications for coverage decisions: Evidence from 32 countries. PharmacoEconomics - Open. 2022;6(3):315–328. 10.1007/S41669-021-00311-5/FIGURES/3 34845671 PMC9043057

[48] Mbau R, Vassall A, Gilson L, Barasa E. Factors influencing institutionalization of health technology assessment in Kenya. BMC Health Serv Res. 2023;23(1):1–14. 10.1186/S12913-023-09673-4/TABLES/3 37349812 PMC10288787

[49] Dwivedi R, Athe R, Pati S, Sahoo KC, Bhattacharya D. Mapping of health technology assessment (HTA) teaching and training initiatives: Landscape for evidence-based policy decisions in India. J Fam Med Prim Care. 2020;9(11):5458. 10.4103/JFMPC.JFMPC_920_20 PMC7842426 33532379

