# Supplementary material for: Health technology assessment (HTA) readiness in Uganda: stakeholder’s perceptions on the potential application of HTA to support national universal health coverage efforts
Source: Int J Technol Assess Health Care. 2023 Oct 31;39(1):e65. doi: 10.1017/S0266462323002635 (PMC11579667; doi:10.1017/S0266462323002635)
Supplement: Mayora et al. supplementary material [file S0266462323002635sup001.docx]

**Supplementary material 1**

Table 1: Affiliations of the key informants included in this study

| **Category** | **Institution** | **Number of key informants (N=30)** | **Percentage (%)** |
| --- | --- | --- | --- |
| **HTA supply side** | Academic institutions (both public and private universities) | 5 | 16.7 |
|  | Government health research departments/institutions | 1 | 3.3 |
|  | Professional Associations in health economics (International Health Economics Association Uganda Chapter) | 1 | 3.3 |
|  | Private research firms (e.g Consultancy firms) | 2 | 6.7 |
|  | Non-Governmental Organizations and Development partners | 4 | 13.3 |
| **Demand side for HTA** | Government ministries (eg MoH and Ministry of Finance) | 8 | 26.7 |
|  | Government departments responsible for procurement and regulation of medicines (e.g National Medical Stores and National Drug Authority) | 2 | 6.7 |
|  | Regional governments | 1 | 3.3 |
|  | Pharmaceutical companies | 1 | 3.3 |
|  | Professional Councils (Medical and Dental Practitioners Council and Allied Health Professionals Council) | 3 | 10.0 |
|  | NGOs and development partners | 2 | 6.7 |
